# Towards Flexible Dielectric Materials with High Dielectric Constant and Low Loss: PVDF Nanocomposites with both Homogenously Dispersed CNTs and Ionic Liquids Nanodomains

**DOI:** 10.3390/polym9110562

**Published:** 2017-10-28

**Authors:** Yanyuan Wang, Chenyang Xing, Jipeng Guan, Yongjin Li

**Affiliations:** College of Material, Chemistry and Chemical Engineering, Hangzhou Normal University, No. 16 Xuelin Road, Hangzhou 310036, China; Yanyuan_W@163.com (Y.W.); xingchenyang319@163.com (C.X.); gj864988@126.com (J.G.)

**Keywords:** poly(vinylidene fluoride) (PVDF), carbon nanotube, ionic liquid, ionic nanoclusters, dielectric performance, flexibility

## Abstract

Flexible dielectric materials with high dielectric constant and low loss have attracted significant attention. In this work, we fabricated novel polymer-based nanocomposites with both homogeneously dispersed conductive nanofillers and ion-conductive nanodomains within a polymer matrix. An unsaturated ionic liquid (IL), 1-vinyl-3-ethylimidazolium tetrafluoroborate ([VEIM][BF_4_]), was first coated on the surface of multi-walled carbon nanotubes (CNTs) by the mechanical grinding. The ILs coated CNTs were then well dispersed in poly(vinylidene fluoride) (PVDF) matrix by melt-blending. The ILs on the surface of CNTs were subsequently grafted onto the PVDF chains by electron beam irradiation (EBI). The formed ILs grafted PVDF (PVDF-*g*-IL) finally aggregated into ionic nanodomains with the size of 20–30 nm in the melt state. Therefore, novel PVDF nanocomposites with both homogenously dispersed CNTs and ionic nanodomains were achieved. Both carbon nanotubes and ionic nanodomains contributed to the enhancement of the dielectric constant of PVDF significantly. At the same time, such homogeneously dispersed CNTs along with the confined ions in the nandomains decreased current leakage effectively and thus led to the low dielectric loss. The final PVDF nanocomposites exhibited high dielectric constant, low dielectric loss and good flexibility, which may be promising for applications in soft/flexible devices.

## 1. Introduction

Dielectric materials have attracted significant attention in terms of their energy storage applications [1,2,3,4]. Among the various physical properties, high dielectric constant, low dielectric loss and high electric breakdown strength have become three important parameters for dielectric materials. Dielectric ceramics materials can fulfill such requirements [4]. However, the inherent brittleness of the inorganic materials limits their applications in soft/flexible devices. Flexibility is of great importance for developing electric charge storage capacitors with miniature, lightweight and ultrathin features. Therefore, extensive investigations have been carried out to make the composites by combining the high-permittivity inorganic [5,6,7,8,9,10,11] or conductive nanofillers [12,13,14,15,16,17,18] with flexible polymers. Among the numerous dielectric polymer composites, poly(vinylidene fluoride)/carbon nanotubes (PVDF/CNTs) nanocomposites have been extensively investigated [19,20,21,22,23]. It has been found that an extremely low loading level of CNTs can significantly improve the dielectric constant of PVDF due to its great dielectric constant and long aspect ratio [24,25]. Although CNTs can enhance the dielectric constant of PVDF at a low levels (below the percolation threshold [19]), their agglomeration nature due to the strong van der Waals interaction and/or π–π interaction between CNTs makes this strategy challenging. The CNTs agglomerates with large domain size and the poor PVDF-CNTs interfaces can inevitably cause serious current leakage and dielectric loss for the PVDF/CNTs nanocomposites. Therefore, the key issue to fabricate high-dielectric-performance PVDF/CNTs composites is still located to disperse CNTs in the PVDF matrix homogeneously.

On the other hand, room temperature ionic liquids (ILs) have recently been used as soft additives to enhance the dielectric constant of PVDF due to their high ion conductivity [26,27,28] and good miscibility with PVDF matrix, as reported in our previous works [29,30,31]. However, the ion movements of ILs in the PVDF/IL blends cause considerable dielectric loss under AC electric filed. Fortunately, the dielectric loss due to the ion movements can be effectively suppressed by chemically grafting the ions onto the PVDF main chains by using electron beam irradiation (EBI) [32,33]. A further reduction in dielectric loss of the PVDF/IL blends can be achieved by confining both cations and anions within IL molecules grafted PVDF (i.e., PVDF-*g*-IL) nanodomains [34,35].

Considering the structure and properties of PVDF/CNTs nanocomposites and the microphase-separated PVDF/IL blends with the PVDF-*g*-IL nanodomains [34,35], it is interesting to fabricate the PVDF nanocomposites with both CNTs and the PVDF-*g*-IL nanodomains. More interestingly, it has been reported that ILs show specific interactions with CNTs and that ILs can help the dispersion of CNTs in polymer matrix [31,36,37]. Therefore, the incorporation of ILs may first improve the dispersion of CNTs and then can microphase separate after a grafting-melting-procedure in the PVDF matrix. Such an idea might lead to the new PVDF nanocomposites with both CNTs and ILs nanodomains, and the nanocomposites may show high dielectric performance and good flexibility. In this study, 1-vinyl-3-ethylimidazolium tetrafluoroborate ([VEIM][BF_4_]) is firstly used to improve the dispersion of CNTs within the PVDF matrix and the cations of the ILs are then chemically grafted onto PVDF chains by electron-beam irradiating PVDF/IL-CNTs films at room temperature in air. A following melting process gives rise to the PVDF nanocomposites with homogeneously dispersed CNTs and organic conductive PVDF-*g*-IL nanodomains in the PVDF matrix. The as-prepared PVDF nanocomposites exhibited significantly enhanced dielectric constant (>100 at 10^3^ Hz) and low loss (<1 at 10^3^ Hz) with only 1 wt% CNTs content. Moreover, the dielectric loss of CNTs can be significantly suppressed by this strategy. Although numerous strategies have been proposed to meet such requirements, such as coating and surface modification of fillers [38,39,40], filler alignment by electrospinning [39] and injection molding [41], the use of hybrid additives [42,43], the modulation of topological structure [44], and the alignment of fillers by cell growth during polymer foaming [45], blend morphology development [46], the present work provides new avenue to fabricate the high performance dielectric polymer materials. 

## 2. Materials and Methods

### 2.1. Materials

PVDF pellets (KF850) were purchased from Kureha Chemicals in Tokyo, Japan, with a *M_w_* of 2.09 × 10^5^ and an *M_w_/M_n_* of 2. The unsaturated ionic liquid (IL), 1-vinyl-3-ethylimidazolium tetrafluoroborate [VEIM][BF_4_], was bought from Center of Greenchemistry and Catalysis, Lanzhou, China and was used as received. Multiwall carbon nanotubes (MWCNTs) were kindly provided by Nikkiso Co. Ltd (Tokyo, Japan). The purity of the MWCNTs is higher than 95%. The diameter of MWCNTs is about 20 nm and the average length ranges from 2 to 10 μm.

### 2.2. Preparation of PVDF/MWCNTs Nanocomposites with IL Nanodomains

The final nanocomposites were prepared by the following steps: (1) preparation of ILs modified CNTs (IL/CNTs)—the MWCNTs were first ground with the ILs at room temperature with various weight ratios, MWCNTs bulky gel was thus prepared and termed IL/CNTs; (2) preparation of PVDF/IL-CNTs nanocomposites—the IL/CNTs were melt compounded with PVDF at 190 °C using a Haake mixer (Haake Polylab QC), (Thermo Fisher Scientific, Waltham, MA, USA) with the screw rotation speed of 50 rpm, the PVDF/IL-CNTs blends were then hot-pressed at 190 °C into films with the thickness of about 300 μm; (3) preparation of EB irradiated PVDF/IL-CNTs nanocomposites—the hot pressed films were irradiated at a dose of 45 kGy in air at room temperature using an electron beam accelerator, the as-irradiated PVDF/IL-CNTs films were thus fabricated and termed ir-PVDF/IL-CNTs (the acceleration energy and beam current were 2.5 MeV and 17 mA, respectively); and (4) preparation of PVDF/CNTs with IL nanodomains—the EBI irradiated PVDF/IL-CNTs nanocomposites were heated to 210 °C for 30 min, followed by a cooling procedure, and the PVDF nanocomposites with IL nanodomains were then prepared; they were termed nano-PVDF/IL-CNTs nanocomposites (for instance, the sample of PVDF/IL-CNTs 100/10-1 meant that the weight ratios of PVDF, IL and CNTs was 100:10:1). 

### 2.3. Morphological Characterization

Field-emission scanning electron microscope (FE-SEM) has been used to characterize the dispersion of MWCNTs in the PVDF matrix. The measurements were carried out using Hitachi S-4800 SEM (HITACHI, Tokyo, Japan) at an accelerating voltage of 5 kV. All the samples were fractured in liquid nitrogen and the fracture surface was then coated with a thin layer of gold before the observation. Transmission electron microscopy (TEM) was also performed using a Hitachi HT-7700 (HITACHI, Tokyo, Japan) operating at an acceleration voltage of 100 kV. The composite samples were ultramicrotomed to a section at −120 °C into a thickness of about 80 nm. The sections were then stained using ruthenium tetroxide (RuO_4_) for 4 h.

### 2.4. Properties Measurements

Differential scanning calorimeter (DSC) with a type of TA-Q2000 (TA Instruments, New Castle, USA) was performed to determine the melt-crystallization temperatures (*T_c_*) of samples. Samples were first heated to 230 °C, which is higher than the equilibrium melting point of PVDF, for 10 min under N_2_ atmosphere to vanish their thermal history. The following cooling down process to −50 °C at a cooling rate of 10 °C/min was recorded. 

The crystal forms of samples were determined by using wide-angle X-ray diffraction (WAXD) with a Bruker D8 type (Bruker, Karlsruhe, Germany). The detective angles of the WAXD experiments were from 5° to 40° at 1°/min. The correlations of crystal-amorphous parts in the PVDF and the IL nanoclusters-PVDF matrix were determined by using small-angle X-ray scattering (SAXS) at 16B beam line in Shanghai Synchrotron Radiation Facility in China. The wavelength of X-ray beam, sample-detector distance and exposed time were 1.24 Å, 1943 mm and 200 s, respectively. The obtained two-dimensional array images were processed with a fit 2D software. Alpha-N high-resolution dielectric analyzer (GmbH Concept 40) (Novocontrol Technologies, Montabaur, Germany) was used to evaluate the dielectric properties of PVDF samples. Circle samples with diameter of 1 mm and thickness of 0.3 mm were sprayed with gold layers on both surfaces before measurements at room temperature. The frequency from 10^ to ^–10^7^ Hz and the AC of 1.0 V were adopted, respectively.

Raman spectra were obtained by using a Bruker raman system (Bruker, Karlsruhe, Germany) with a Senterra R200 type. The wavelength of laser was 785 nm and at least three different locations were measured on the samples. The final reported data was averaged.

Thermogravimetric analysis (TGA) with a TA-Q500 type (TA Instruments, New Castle, DE, USA) was used to investigate the thermal stability of samples. Each sample with about 5 mg was heated from room temperature to 650 °C with a heating rate of 10 °C/min under a continuously high purity N_2_ atmosphere. Note that the excess ILs on the surface of CNTs was removed by resolving the CNTs/IL sample in methanol (CH_3_OH) and then by a centrifugation treatment before measurement. The CH_3_OH was the good solvent of IL here. 

Mechanical properties of samples were determined by using a universal material testing with an Instron-5966 model (Instron, Norwood, MA, USA). The samples were cut into dumbbell shape before measurements. The stretched speed was 10 mm/min and a fixed gauge length was 18 mm, respectively. An averaged value was reported after at least three measurements. 

Direct current (DC) electrical properties of samples were evaluated by measuring their surface resistivity (Rs) with an ultrahigh resistivity meter (MCP-HT450) (Mitsubishi, Nagasaki, Japan) at room temperature. The applied DC voltage was 10.0 V. An average value of Rs was reported after at least five measurements on the samples.

## 3. Results

### 3.1. The ILs Coated CNTs and Their Homogeneous Dispersion in PVDF Matrix

**Figure 1:**
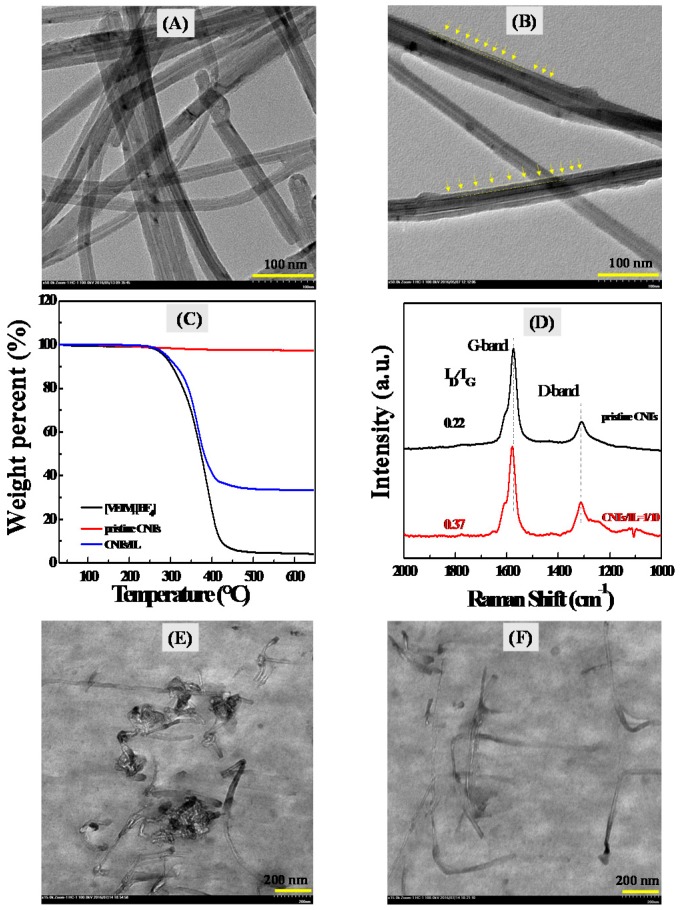
(**A**) Transmission electron microscopy (TEM) image of pristine carbon nanotubes (CNTs) dispersed in the water; (**B**) TEM image of ionic liquids (ILs) coated CNTs dispersed in the water; (**C**) Thermogravimetric analysis (TGA) curves of pristine CNTs, pure ILs and ILs modified CNTs, respectively; (**D**) Raman spectra of pristine CNTs and IL coated CNTs (i.e., CNTs/IL = 1/10); (**E**) TEM image of PVDF/CNTs (100/1) composite; (**F**) TEM image of PVDF/ IL-CNTs (100/10-1) nanocomposites.

A good dispersion state of CNTs within PVDF matrix can decrease their current leakage and dielectric loss effectively. ILs have been demonstrated to modify CNTs via a possible cation-π interaction [47,48,49]. In view of this, CNTs were first grounded with ILs and the corresponding IL/CNTs bulky gel was formed. As shown in Figure 1A, pristine CNTs have large agglomeration in size and their bundles entangle each other. This can be attributed to the strong van der Waals interaction and/or π–π interaction between CNTs. After treatment for CNTs by ILs, CNTs are debundled by ILs and single CNTs can be observed (Figure 1B). Besides, a clear organic layer of IL molecules can be observed in the ILs modified CNTs hybrids. A further confirmation of ILs onto the surface of CNTs is found, according to the TGA curves, as shown in Figure 1C. A decrease of weight percent in IL/CNTs hybrids is in fact the decomposition of ILs that coated on the surface of CNTs by the possible cation-π interaction during heating.

Note that the excess IL molecules in the IL/CNTs hybrids used in the TGA measurements have been removed by centrifugation in advance, as shown in the Experimental Section (2.4). The interaction of CNTs with ILs can be directly characterized by Raman spectra in Figure 1D. Two peaks in Raman spectra for pristine CNTs can be assigned to G-band and D-band. The treatment by ILs leads to a red shift of G-band and a variation of ratio of *I_G_/I_D_*, indicating that the electric properties of surface of CNTs are affected by IL-layers coating. This phenomenon is also reported in other literatures [50].

The IL-coated CNTs were directly dispersed in PVDF matrix by melt blending. Figure 1E,F show the TEM images of PVDF composites with pristine (unmodified) CNTs and IL-coated CNTs, respectively. It is clear that the pristine CNTs agglomerate in the PVDF matrix (Figure 1E), while the significantly improved CNTs dispersion can be observed after the coating of CNTs by ILs (Figure 1F). This can be attributed to the bridging effects of ILs that couples the CNTs with the PVDF matrix [31].

### 3.2. The Irradiation Induced In Situ Grafting of ILs onto PVDF and the Following Phase Separation of PVDF-g-IL

**Figure 2:**
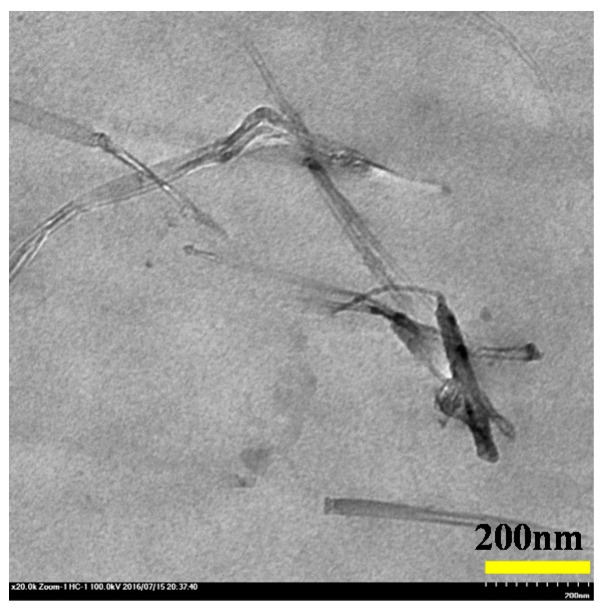
TEM image of as-irradiated poly(vinylidene fluoride) (PVDF)/IL-CNTs (100/10-1).

The above results confirm that the ILs benefit the dispersion of CNTs in the PVDF matrix. However, ILs can generate considerable dielectric loss by their both cations and anions movements in AC electric field [32,33,34]. This obviously impairs the dielectric performance of PVDF/IL-CNTs composites. In order to reduce the dielectric loss induced by the movements of ions of ILs in the nanocomposites, we try to chemically bonding the cations of ILs onto the PVDF molecular chains. The melt-prepared composites films were then exposed upon the electron beam irradiation at room temperature. We have previously reported the chemically grafting of the ILs onto the PVDF by electron beam irradiation in binary PVDF/IL blends [32,33,34]. In that binary system, ILs were located in the amorphous region of PVDF at room temperature [34,35]. Therefore, the high energy electrons from electron beam generators induce free radicals by knocking out the H and/or F atoms from the PVDF backbones and the double bonds of the cations in ILs [32,51]. The cations of ILs were then grafted locally onto the PVDF chains through coupling of these free radicals [35]. In the present case, the ILs are mainly located at the interface between the CNTs and the PVDF matrix. The irradiation will lead to the ILs at the interface to graft onto the PVDF molecular chains at the room temperature. It should be noted that some excess ILs may also locate at the amorphous region of PVDF during the melt mixing due to the miscibility between PVDF and ILs. Thus, those ILs will also be grafted onto the PVDF amorphous region, similar to the behavior in the PVDF/ILs binary systems [32,34,35]. Figure 2 shows the TEM image of the as-irradiated PVDF/IL-CNTs nanocomposites. It is seen that the morphology of the as-irradiated nanocomposites is almost the same with that of the melt-mixed nanocomposites before the irradiation (Figure 1F). This is rational because the irradiation was carried out at room temperature and the chemical grafting occurs locally. No morphology changes can be expected when the samples were irradiated at room temperature [32].

**Figure 3:**
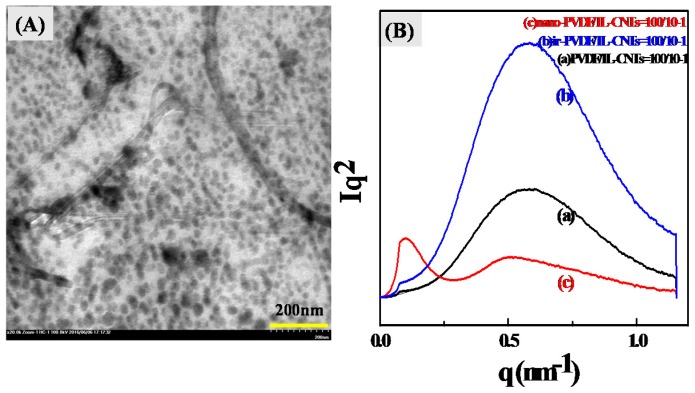
(**A**) TEM image of nano-PVDF/IL-CNTs (100/10-1) and (**B**) Small-angle X-ray scattering (SAXS) patterns of PVDF/IL-CNTs (100/10-1), irradiated-PVDF/IL-CNTs (100/10-1) and nano-PVDF/IL-CNTs (100/10-1) samples.

The as-irradiated PVDF/IL-CNTs were then heated to 210 °C and kept at there for 30 min, followed by the cooling down to the room temperature. The samples were then evaluated by TEM and the image is shown in Figure 3A. 

Totally different from the structure of the as-irradiated sample before the melting, numerous black domains with the size of around 20–30 nm are observed, except for the CNTs in the matrix. We can also observe some nanodomains which are adhering to the surface of the CNTs. The formation of the nanodomains during the melting can also be confirmed by the SAXS measurements, as shown in Figure 3B. Only one scattering peak was observed for the PVDF/IL-CNTs and as-irradiated PVDF/IL-CNTs at the *q* of 0.58 nm^−1^. This peak originates from the lamellar structure of PVDF matrix. The density difference between the amorphous region and the crystalline region contributes to such scattering. In contrast, the nano-PVDF/IL-CNTs (100/10-1) sample displays a totally distinct scattering pattern. Two strong scattering peaks at *q* = 0.09 nm^−1^ and *q* = 0.53 nm^−1^, respectively, are observed. Obviously, the scattering peak at *q* = 0.53 nm^−1^ originates from the lamellar structure in the PVDF crystals. The other peak at *q* = 0.09 nm^−1^ originates from the nanodomains in the PVDF matrix. It is considered that the melting processing of the as-irradiated sample leads to microphase separation of PVDF-*g*-IL chains from PVDF matrix, similar to the PVDF/IL binary systems [34,35].

**Figure 4:**
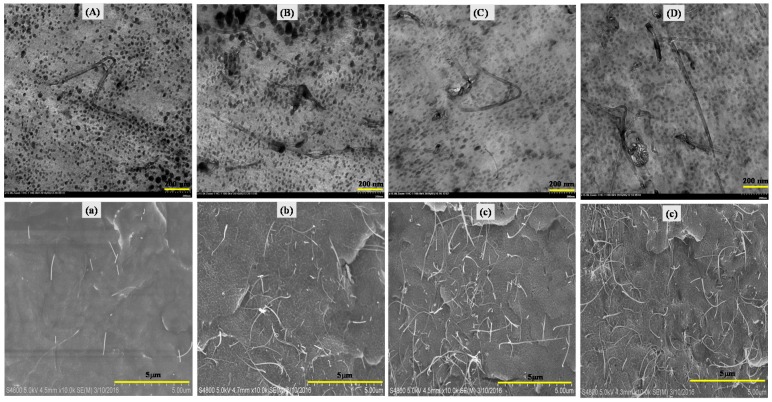
TEM and SEM images of nano-PVDF/IL-CNTs samples with various content of CNTs: (**A**) and (**a**): 100/10-0.1; (**B**) and (**b**): 100/10-0.5; (**C**) and (**c**): 100/10-1; (**D**) and (**d**) 100/10-2, respectively.

Figure 4 shows the TEM and SEM images of nano-PVDF/IL-CNTs samples with various IL/CNTs ratios. The IL content keeps constant with changing the CNTs loadings in the final nanocomposites. It is seen that the CNTs in the all samples are almost perfectly dispersed in the PVDF matrix. The microphase separation in the melt does not induce the re-aggregation of CNTs. This is attributed to the long aspect ratio of CNTs and the high viscosity of the matrix melt. As a dielectric material, the CNTs content within PVDF matrix should be less than their percolation threshold [19] because the formation of conductive pathway in the PVDF is not accessible to the dielectric materials. With increasing the CNTs contents, the number of conductive pathway increases. On the other hand, it is clear that all the samples have the nanodomains and no significant difference could be observed for the nanodomains with varying the CNTs loadings. It is also seen that CNTs form a conductive networks at the loading of 2 wt% CNTs, which means this sample might not be suitable for fabricating dielectric materials.

### 3.3. The Crystallization Behaviors and the Crystal Form in the Nano-PVDF/IL-CNTs

**Figure 5:**
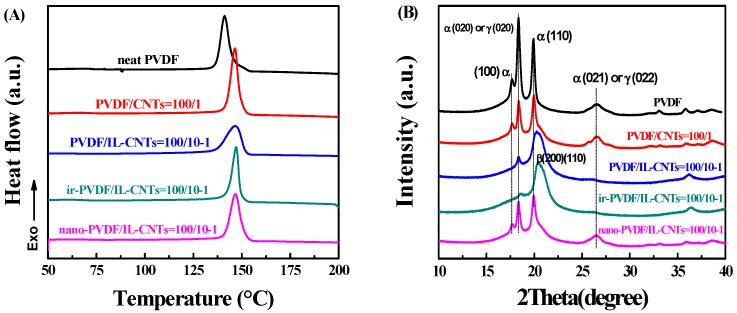
Characterization of crystallization behaviors of nano-PVDF/IL-CNTs (100/10-1) nanocomposite in comparison with its counterparts, including neat PVDF, PVDF/CNTs (100/1), PVDF/IL-CNTs (100/10-1) and ir-PVDF/IL-CNTs (100/10-1). (**A**): Differential scanning calorimeter (DSC) cooling curves with a 10 °C /min cooling rate; (**B**) Wide-angle X-ray diffraction (WAXD) patterns in the range of 10–40° with a scanning rate of 1°/min.

Figure 5 shows the crystallization behaviors of neat PVDF, binary blends PVDF/CNTs (100/1), ternary PVDF/IL-CNTs (100/10-1), as-irradiated PVDF/IL-CNTs (100/10-1) and nano-PVDF/IL-CNTs (100/10-1) composites, respectively. In Figure 5A, neat PVDF shows a crystallization temperature (*T_c_*) at 141.1 °C when cooling down from the melt at a cooling rate of 10 °C/min. However, for the other three samples with CNTs, the *T_c_* increased to about 146.3 °C, indicating the nucleation effects of the CNTs. It is further observed that the crystallization peak of PVDF/IL-CNTs is wider than those of the PVDF/CNTs sample and nano-PVDF/IL-CNTs. ILs are miscible with PVDF matrix, so the simple addition of ILs leads to the depression of the *T_c_* in binary PVDF/ILs [31]. Therefore, the IL-coated CNTs induce higher *T_c_* with the wider crystallization peak. In other words, the ILs at the interface between the CNTs and the PVDF impede the CNTs nucleation effects partially. On the other hand, both the PVDF/CNTs and nano-PVDF/IL-CNTs show significant nucleation effects for the matrix PVDF with a very sharp crystallization peak. The high nucleation effects of CNTs in nano-PVDF/IL-CNTs possibly indicate ILs peeling off from the interface and CNTs directly nucleate the crystallization of PVDF.

PVDF has various types of crystal forms and the polar β or γ crystals exhibit ferroelectric properties. Figure 5B shows XRD patterns of the neat PVDF, PVDF/CNTs (100/1), PVDF/IL-CNTs (100/10-1), as-irradiated PVDF/IL-CNTs (100/10-1) and nano-PVDF/IL-CNTs (100/10-1). Neat PVDF shows the typical nonpolar α phase with the diffractions at 2θ = 17.7°, 18.4°, 20.0° and 26.6°, corresponding to the (100), (020), (110) and (021), respectively. The simple addition of unmodified CNTs does not change the crystal forms of the matrix PVDF. Although literatures reported the CNTs induced crystal form transitions of PVDF [44], the aggregation of CNTs provides very limit contact of CNTs with molecular chains of PVDF. However, significant difference was observed with the addition of ILs coated CNTs. PVDF crystallizes into mainly the polar β phase. The almost zigzag conformation of PVDF was induced due to the specific interactions between >CF_2_ of PVDF with the planar cationic imidazolium ring wrapped on the CNTs surface; thus, nucleation in polar crystals (β and γ forms) lattice is achieved and polar crystals are obtained by subsequent crystal growth from the nuclei [31]. The electron beam irradiation at room temperature locally grafts ILs onto the PVDF chains and this does not affect the crystal forms of PVDF [32,33,34]. Therefore, the as-irradiated PVDF/IL-CNTs shows almost same WAXD pattern as the PVDF/IL-CNTs and the PVDF is mainly polar crystal forms. However, the melting of the irradiated sample induces the microphase separation of PVDF-*g*-IL and no specific interaction occurs between cations with CF_2_ in the melt. The nonpolar PVDF α crystals were obtained when cooling down, as similar to that of neat PVDF. This indicates again that the possible peeling off of ILs from CNTs during the microphase separation.

### 3.4. Physical Properties of the PVDF Nanocomposites with Both CNTs and Nanodomains

#### 3.4.1. Electrical Conductivity of the PVDF Nanocomposites

**Figure 6:**
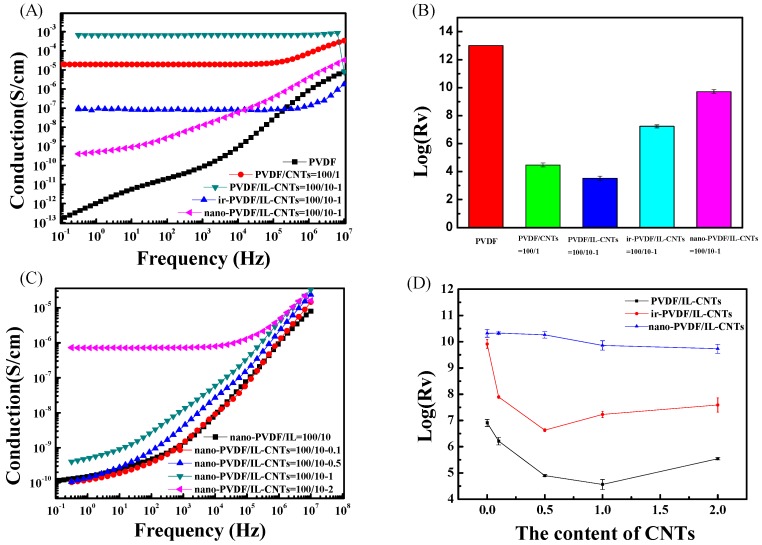
Electrical properties of PVDF and PVDF-based samples. (**A**,**B**) are AC conductivity and surface resistivity (Rs) of typical samples including neat PVDF, PVDF/CNTs (100/1), PVDF/IL-CNTs (100/10-1), ir-PVDF/IL-CNTs (100/10-1) and nano-PVDF/IL-CNTs (100/10-1), respectively; (**C**) AC conductivity of nano-PVDF/IL-CNTs samples with various CNTs loading levels; (**D**) Rv values of three typical systems, including PVDF/IL-CNTs, as-irradiated PVDF/IL-CNTs and nano-PVDF/IL-CNTs with different CNTs contents.

Figure 6 shows AC electrical conductivity (Figure 6A) and DC electrical resistivity (Figure 6B) of neat PVDF and PVDF-based composites. CNTs nanofillers reduce the surface resistivity (R_s_) of PVDF in PVDF/CNTs (100/1) because of the excellent conductivity of CNTs. The PVDF/IL-CNTs (100/10-1) composites have higher conductivity because of the improved CNTs dispersion and also the good ionic conductivity of IL itself. The grafting of ILs limits the movement of ions, so the conductivity of as-irradiated PVDF/IL-CNTs is lower than that of the PVDF/IL-CNTs. However, the nano-PVDF/IL-CNTs show lowest electrical conductivity in the all samples due to the confinement of the ions in the nanodomains. Figure 6C,D shows the electrical conductivity of the nano-PVDF/IL-CNTs with various amount of CNTs. Obviously, the increasing of CNTs loadings did not affect the conductivity of nano-PVDF/IL-CNTs samples. It can be attributed to the well dispersed CNTs and the confined ions. 

#### 3.4.2. Dielectric Performance of the PVDF Nanocomposites

**Figure 7:**
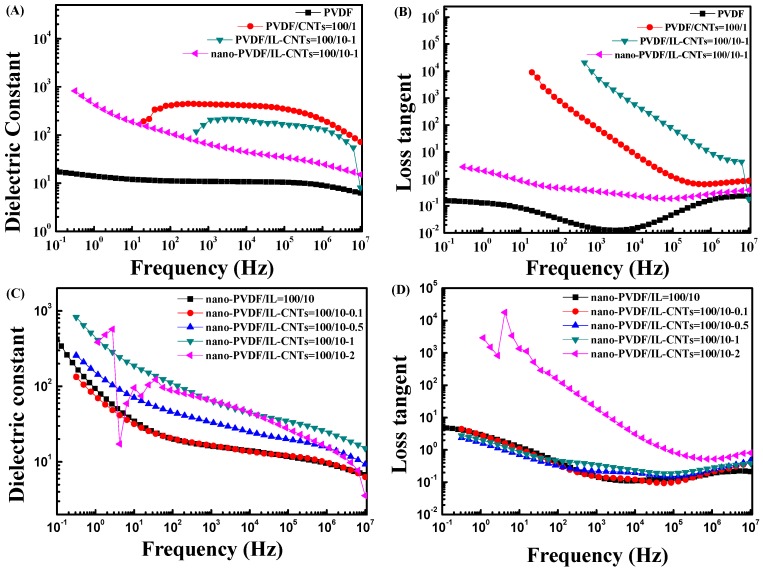
Frequency dependency of dielectric constant (**A**,**C**) and loss tangent (**B**,**D**) of samples including neat PVDF, PVDF/CNTs (100/1), PVDF/IL-CNTs (100/10-1), as-irradiated PVDF/IL-CNTs (100/10-1) and nano-PVDF/IL-CNTs with various CNTs contents.

Figure 7A,B shows the dielectric constant and the loss for neat PVDF and PVDF based composites. As also shown in Figure 6, the PVDF/CNTs (100/1) and PVDF/IL-CNTs (100/10-1) have high electrical conductivity, and both of the two samples exhibit the dielectric behaviors of conductive materials in Figure 7A,B. Therefore, no dielectric permittivity can be recorded at the low frequency for the conductive composites. Similar phenomena can also be observed for the nano-PVDF/IL-CNTs (100/10-2) (Figure 7C,D). The high CNTs loading leads to the conductive nature of the sample. However, for the microphase separated ternary nanocomposites with the CNTs loadings lower than the percolation threshold, they exhibit higher dielectric constant than the neat PVDF. According to the Maxwell-Wagner-Sillars (MWS) effect [52,53], charges can be accumulated at the interface when electric current flows across the PVDF/CNTs and the PVDF/nanodomains interfaces. This largely enhanced the average electric field and thus the permittivity of PVDF matrix. However, poor compatibility of PVDF with pristine CNTs caused relatively larger CNTs agglomeration, which increased leakage current and induced high dielectric loss. Therefore, the simply melt mixed PVDF/CNTs (100/1) do not show the good dielectric performance. In contrast, the nano-PVDF/IL-CNTs (100/10-1) have the excellent dielectric performance with the high dielectric constant and depressed dielectric loss.

Figure 7C,D show the effects of different CNTs content on the dielectric constant (Figure 7C) and loss tangent (Figure 7D) of microphase separated PVDF ternary nanocomposites. Obviously, too much CNTs loading (2 wt%) leads to the conductive nature of the nano-PVDF/IL-CNTs (100/10-2) sample. However, it is very interesting to find that, in Figure 7C, as the content of CNTs increases, the permittivity of nanocomposite increases significantly in the CNT loadings ranging from 0.1 to 1%. At the same time, all the samples have the almost same dielectric loss in Figure 7D. Specifically, the dielectric constant and loss of the nano-PVDF/IL-CNTs (100/10-1) are 180 and 0.81 at 10^2^ Hz, respectively. The values are 19 and 0.82 for the nano-PVDF/IL-CNTs (100/10-0.1) sample. This means that we can enhance the dielectric constant, but keep almost constant loss simultaneously with increasing the CNT loadings. 

#### 3.4.3. Mechanical Properties of the PVDF Nanocomposites

**Figure 8:**
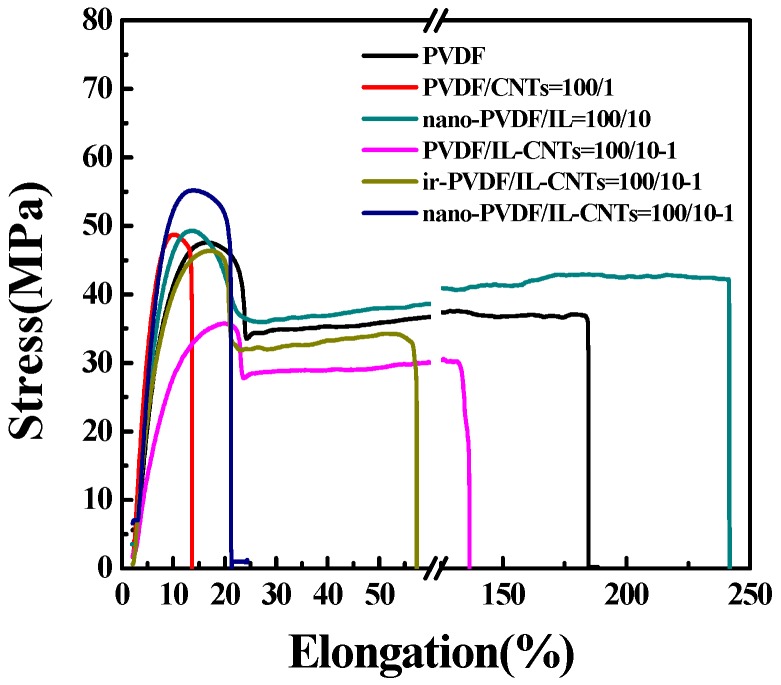
Strain-stress curves of neat PVDF, PVDF/CNTs (100/1), PVDF/IL-CNTs (100/10-1), ir-PVDF/IL-CNTs (100/10-1), and nano-PVDF/IL-CNTs (100/10-1).

Figure 8 shows the strain-stress curves of the neat PVDF and its nanocomposites with ILs and CNTs. It is clear that the pristine CNTs lead to slightly increased modulus and strength, but with drastically decreased elongation at break. The modified CNTs by ILs can be well dispersed in the PVDF matrix and the interface is improved by the ILs, so PVDF/IL-CNTs (100//10-1) have much higher stretchability than the PVDF/CNTs (100/1) composites. However, the ILs plasticize PVDF simultaneously and the strength of the nanocomposites decreases significantly. The irradiation induces the fixation of the small molecules of ILs and the plasticizer effects of ILs are much depressed. The ir-PVDF/IL-CNTs show enhanced yielding strength and decreased elongation at break when compared with the PVDF/IL-CNTs. The microphase separation leads to the aggregation of PVDF-*g*-IL. This means that the PVDF-*g*-IL located at the interface between the CNTs and matrix aggregate together to form nanodomains, so the interface was weakened. Therefore, we observed much decreased elongation at break of about 20%. It should be noted that the nano-PVDF/IL-CNTs (100/10-1) exhibited higher modulus than the nano-PVDF/CNTs (100/10) and show pretty good stretchability of 20%, which is significantly higher than that of the dielectric ceramics.

## 4. Discussion

**Figure 9:**
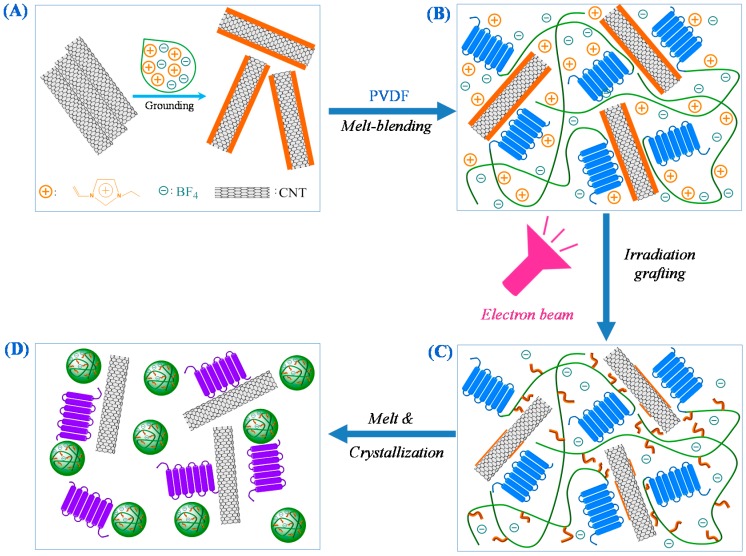
Schematic diagrams for the formation of ionic nanoclusters and CNTs structures in nano-PVDF/IL-CNTs composites. (**A**) First grounding of CNTs with IL resulted in IL-layer-coated CNTs; (**B**) melt-blending PVDF with such IL-layer-coated CNTs fabricated PVDF/IL-CNTs nanocomposites with homogeneously dispersed CNTs; (**C**) The PVDF/IL-CNTs nanocomposites then exposed upon electron beam irradiated (EBI) at room temperature in the air, whereas IL molecules in-suit grafted onto chains of PVDF; (**D**) the as-irradiated PVDF/IL-CNTs samples were heated to 210 °C and held there for 30 min, and microphase separation of IL grafted PVDF (PVDF-*g*-IL) chains occurred in the melt. A following cooling procedure led to the nano-PVDF/IL-CNTs composites with PVDF-*g*-IL nanodomains (i.e., IL nanoclusters).

**Figure 10:**
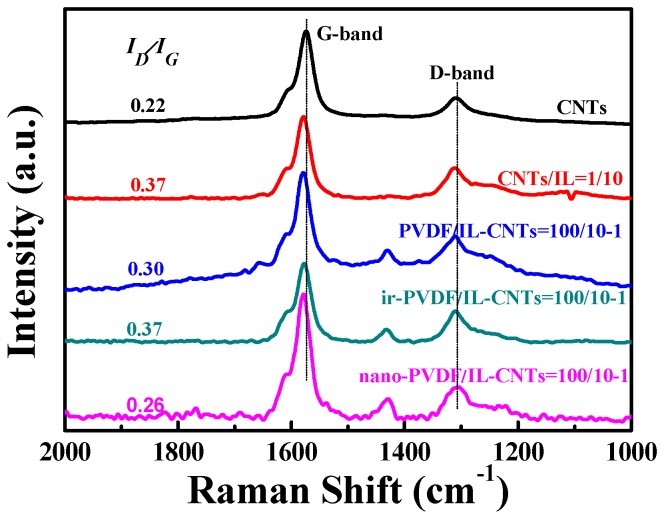
Raman spectra of pristine CNTs, IL coated CNTs (CNTs/IL = 1/10), PVDF/IL-CNTs (100/10-1), as-irradiated PVDF/IL-CNTs (100/10-1) and nano-PVDF/IL-CNTs (100/10-1) samples, respectively.

For dielectric materials, increased permittivity is usually accompanied with enhanced dielectric loss. It is very interesting to find in the present work that the nano-PVDF/IL-CNTs samples show enhanced dielectric constant with increasing CNTs loading but keep almost the constant dielectric loss, in the range of less than 2 wt% CNTs. The unique behavior can be attributed to the double formation of the nanophases in the PVDF matrix, i.e., the homogeneously dispersed CNTs and PVDF-*g*-IL nanodomains. It is important to elucidate the formation of the double nanophases structure by the combination of the ILs modification of CNTs with the irradiation process. Figure 9 depicts the schematic diagrams of the formation of the double nanophases. ILs were first coated on the surface of CNTs due to the specific cation-π interactions by grinding (Figure 9A), which is confirmed by the enhanced D-band to G-band intensity ratio after the coating compared with the pristine CNTs in Raman spectra (Figure 10). The surface-coated CNTs can be homogeneously dispersed in PVDF matrix (Figure 9B). This is attributed to the cation-CF_2_ interactions between the PVDF and ILs [31]. Therefore, ILs significantly improve the dispersion of CNTs in the PVDF matrix, which leads to the reduction of the current leakage under the AC electric field during the dielectric measurements. In this stage, the ILs are mainly still located on the surface of CNTs, that is, at the interface, as evidenced by the almost no intensity ratio change compared with that of the IL-coated CNTs samples. However, the cations and anions in ILs in the simply blended PVDF/IL-CNTs are mobile and easily move under the electric field, therefore, we still observed high dielectric loss. The electron beam irradiation induces the grafting of the cations with the double bonds onto the PVDF chains (Figure 9C). The irradiation was carried out at room temperature, so the ILs grafting occurs locally and large amount of IL at the interface are chemically bonded onto the PVDF. The melting of the as-irradiated sample leads to the microphase separation of PVDF-*g*-IL from the PVDF matrix (Figure 9D). The formation of the nanodomains of PVDF-*g*-IL can be attributed to the ionic interactions in the melt state. The molecular chains are much dynamic and the ionic interactions lead to the PVDF-*g*-IL nanoclusters and therefore numerous nanodomains were observed in the PVDF matrix (as shown in Figure 3 and Figure 4). Not only the cations grafted onto the PVDF were aggregated in the nanodomains, but also the anions without chemically grafted were confined within the nanodomains simultaneously due to the ionic interactions between the cations and anions [35]. In other words, the PVDF-*g*-IL peels off from the interface between the CNTs and PVDF matrix in the melt state. It should be mentioned that the ILs peeling off from the surface of CNTs does not induce the re-aggregation of CNTs. It is rational that the CNTs have long aspect ratio and the aggregations in the viscous PVDF matrix take long time. The peeling off of ILs from the CNTs can be confirmed direct by the Raman spectra measurements (Figure 10). It is observed that the D-band and G-band intensity ratio of nano-PVDF/IL-CNTs is lower than that of the PVDF/IL-CNTs. Therefore, we can achieve a novel nanocomposite with both homogeneously dispersed CNTs and the PVDF-*g*-IL nanoclusters.

## 5. Conclusions

In this study, a novel nano-PVDF/IL-CNTs composite with both homogeneously dispersed CNTs and ionic nanodomains in the PVDF matrix was fabricated. The processes included surface coating, melt blending, electron beam irradiation (EBI) and microphase separation. The ILs first improved the dispersion of CNTs in the PVDF matrix and then were grafted onto the chains of PVDF by the electron beam irradiation. With the following heating, the PVDF-*g*-IL aggregated into the ionic nanodomains and separated from the ungrafted PVDF matrix. The homogeneously dispersed CNTs and the confined cations and anions in the nanodomains depressed the dielectric loss significantly. Therefore, the nanocomposites exhibit high dielectric constant and low dielectric loss, with good flexibility.
